# Effect of a 5-aminolevulinic acid gel and 660 nm red LED light on human oral osteoblasts: a preliminary in vitro study

**DOI:** 10.1007/s10103-022-03651-8

**Published:** 2022-10-04

**Authors:** Tania Vanessa Pierfelice, Emira D’Amico, Giovanna Iezzi, Morena Petrini, Valeria Schiavone, Manuela Santalucia, Assunta Pandolfi, Camillo D’Arcangelo, Adriano Piattelli, Natalia Di Pietro

**Affiliations:** 1 Department of Medical, Oral and Biotechnological Sciences, University G. d’Annunzio of Chieti-Pescara, Via dei Vestini 31, 66013 Chieti, Italy; 2 Center for Advanced Studies and Technology-CAST (Ex CeSI-MeT), University G. d’Annunzio of Chieti-Pescara, 66013 Chieti, Italy; 3 School of Dentistry, Saint Camillus International, University of Health and Medical Sciences, Via di Sant’Alessandro 8, 00131 Rome, Italy; 4 Dental School, University of Belgrade, Belgrade, Serbia; 5 Fondazione Villa Serena Per La Ricerca, 65013 Città Sant’Angelo, Italy; 6 Casa Di Cura Villa Serena del Dott. L. Petruzzi, 65013 Città Sant’Angelo, Italy

**Keywords:** 5-Delta aminolevulinic acid, Photodynamic therapy, Osteoblasts, Protoporphyrin, Periodontitis, Light-emitting diode

## Abstract

This study aimed to evaluate the effects of a new photodynamic protocol (ALAD-PDT) on primary human osteoblasts (hOBs). The ALAD-PDT protocol consists of a heat-sensitive gel with 5% 5-delta aminolevulinic acid commercialized as Aladent (ALAD), combined with 630 nm LED. For this purpose, the hOBs, explanted from human mandible bone fragments, were used and treated with different ALAD concentrations (10%, 50%, 100% v/v) incubated for 45 min and immediately afterwards irradiated with a 630 nm LED device for 7 min. The untreated and unirradiated cells were considered control (CTRL). The cellular accumulation of the photosensitizer protoporphyrin IX (PpIX), the proliferation, the alkaline phosphatase (ALP) activity, and the calcium deposition were assessed. All concentrations (10, 50, 100%) determined a significant increment of PpIX immediately after 45 min of incubation (0 h) with the highest peak by ALAD (100%). The consequent 7 min of light irradiation caused a slight decrease in PpIX. At 48 h and 72 h, any increment of PpIX was observed. The concentration 100% associated with LED significantly increased hOB proliferation at 48 h (+ 46.83%) and 72 h (+ 127.75%). The 50% and 100% concentrations in combination to the red light also stimulated the ALP activity, + 12.910% and + 14.014% respectively. The concentration 100% with and without LED was selected for the assessment of calcium deposition. After LED irradiation, a significant increase in calcium deposition was observed and quantified (+ 72.33%). In conclusion, the ALAD-PDT enhanced proliferation, the ALP activity, and mineralized deposition of human oral osteoblasts, highlighting a promising potential for bone tissue regeneration.

## Introduction

Light-emitting diodes (LED) and LASER represent valid tools to provide photoinactivation in vitro and in vivo [1–4]. 880 nm LED light provided a significant reduction of planktonic and biofilm of *Enterococcus faecalis* and *Pseudomonas aeruginosa* [1–4]. However, the efficacy of the photoinactivation was dependent on the presence of endogenous photosensitizers, such as protoporphyrin IX (PpIX), inside the bacteria [1–3]. Indeed, the photodynamic therapy (PDT) mechanism implies that the used photosensitizer molecule (PS) must be photoactivated by a light at specific wavelengths to induce its excited state that led to generate the reactive oxygen species (ROS) [5]. An important aspect is that the photosensitizer such as PpIX is selectively accumulated more in abnormal or infected cells, without causing any damages to the healthy tissues [6, 7]. Egli RJ and co-workers evidenced how PpIX differently accumulated in different cell types [8]. For its characteristics, PDT can be a potential strategy in dental field, considering that periodontitis and peri-implantitis are bacterial inflammatory processes that promote the progressive bone loss, a condition difficult to solve [9, 10]. Data from randomized clinical trials indicated that antibacterial PDT (aPDT) performed with a diode laser at 660 nm in combination with photosynthesizers such as phenothiazine chloride, methylene blue, or toluidine blue supported non-surgical periodontal treatments leading to significant improvements in all the investigated clinical parameters (PPD, BOP, CAL) [11, 12]. Many studies conducted in the past confirm the effectiveness of the aPDT treatment using 5-delta aminolevulinic acid (5-ala) as the precursor of the photosensitizer PpIX in the heme biosynthesis [12–15] which have encouraged the production of a sol–gel commercialized as ALADENT (ALAD), specially formulated to deliver this active compound 5-ala at 5% [16–18]. A very appreciable aspect of this gel is its temperature-dependent state transition formulation based on a mixture of poloxamers that ensures the stability of the active ingredient [19, 20]. Indeed, this 5% 5-ala thermolabile formulation is liquid and it is converted to gel with a temperature higher than 28 °C. The subsequent conversion into a gel leads to a controlled spreading of the active component and facilitates its topical administration. The presence of poloxamers improves the muco-adhesion properties of ALAD, making it suitable for skin treatments [21]. Recent in vitro and in vivo studies have shown the capability of the ALAD-PDT protocol to inactivate pathogens involved in periodontal disease [16, 17]. The 5-ala acts as a pro-drug being a precursor of the endogenous photosensitizer PpIX that, unlike other substances such as methylene blue and toluidine blue, has also proved effectiveness against oral bacterial and fungal biofilm [16–18]. These studies demonstrated that the drug-light interval time (45 min + 7 min) is enough to treat oral infections by killing microbes, and provides the bacterial load reduction, together with the reduction of periodontal soft tissue inflammatory parameters, such as PD, CAL, and BOP [4, 16, 22, 23]. Despite the proven strong antibacterial and antifungal effect of ALAD-PDT, which makes it an ideal product for treating periodontitis and peri-implantitis, there is no information regarding of ALAD-PDT potential effects on human cells. Literature poorly provides evidence on the effects of the PDT on healthy cells. Egli et al. revealed cytotoxicity of PDT in different human cell lines, and Bastian et al. obtained similar results in porcine osteoblasts and chondrocytes. Both authors applied the same PDT protocol also used as an anticancer strategy, in terms of time (4 h) and in terms of high light dose [24, 25]. A different approach has been taken by Kushibiki et al. who use PDT doses lower than those conventionally used for cancer treatment and demonstrated the photochemical promotion of mouse and rat osteoblast cell differentiation via ROS production [26]. However, these aforementioned protocols had incubation intervals too high considering that the objective of clinicians is to reduce the working time and consequently also the patient’s compliance. In ALAD-PDT, the incubation and time of irradiation were reduced to 45 min and to 7 min that are lower with respect to the PDT protocols investigated in the cited articles [4, 16, 22, 23]. Based on these previous studies, the ALAD-PDT, until now tested just as antibacterial and antifungal strategy consisting of the thermo-gel containing 5% of 5-ala irradiated by the red LED light at 630 nm, was here investigated to verify if it may promote osteogenic effects. Hence, the purpose of this study was to explore the effects of ALAD gel at different concentrations applied for 45 min, in combination with 7 min of 630 nm red LED light (ALAD-PDT) on human oral osteoblasts.

## Materials and methods

### ALADENT gel

In this study, a thermosensitive gel containing 5% of delta aminolevulinic acid (5-ala), branded as ALADENT (ALAD), commercialized by ALPHA Strumenti s.r.l (Melzo-MI, Italy) has been utilized. This thermosetting product is protected by a patent (PCT/IB2018/060368, 12.19.2018) and remains liquid at temperatures below 28 °C becoming gel at higher temperatures.

### Light device and parameters of irradiation

The light source used is AlGaAs power LED device TL-01(ALPHA Strumenti, Italy) that is a single emitted LED with 6 mm diameter. This 630 nm ± 10 nm LED device FHWM nm emits a red light visible to eyes. During the experimental protocols, the LED handpiece stood in a perpendicular way to the wells, and the illumination arrived from the top at 0.5 mm of distance as illustrated in a previous study [23] The exit and surface irradiance are 380 mW/cm^2^ and the total specific dose is 23 J/cm^2^ for each minute of irradiation, as previously described by Radunovic M et al. [16]. All irradiation procedures were performed in the dark condition under a laminar flow hood.

### Experimental design

A previous Radunovic’s study [16] showed that ALAD gel in combination with 630 nm LED was able to reduce bacteria load already at lower concentrations of 10 and 50% (v/v); thus, in this study the same dilutions of the commercial gel were introduced. For the experiments, the cells were seeded and after 24 h of culture were incubated for 45 min with increasing concentrations (10%, 50%, 100%) (v/v) of ALAD in a serum-free medium at 37 °C. Then, the following experimental conditions were performed: CTRL were untreated (without ALAD) and unexposed (without LED irradiation) cells; 100-ALAD were cells treated with ALAD gel at 100% without LED irradiation; 0-ALAD-PDT were cells exposed to 7 min of LED light irradiation alone without ALAD addition; 10-ALAD-PDT were cells treated with ALAD gel (10%) and exposed to LED for 7 min; 50-ALAD-PDT were cells treated with ALD gel (50%) and exposed to LED for 7 min; 100-ALAD-PDT were cells treated with ALAD gel (100%) and exposed to LED for 7 min. Subsequently, the time-dependent effects of ALAD-PDT on PpIX cell accumulation, proliferation, alkaline phosphatase (ALP) activity, and calcium deposition were assessed. All experiments were performed in triplicate employing different cell strains every time.

### Primary culture of human oral osteoblasts (hOBs)

Following a protocol approved by the Ethics Committee of the University of Chieti-Pescara (reference number: BONEISTO N. 22 10.07.2021), hOBs were obtained from human mandible bone fragments of n° 12 volunteers managed at the dental clinic of the G. D’Annunzio University. The bone fragments underwent three enzymatic digestions at 37 °C for 20, 30, and 60 min utilizing a solution consisting of collagenase type 1A (Sigma-Aldrich, St. Louis, MO, USA) and trypsin–EDTA 0.25% (Sigma-Aldrich) dissolved in Dulbecco’s Modified Eagle’s Medium (DMEM, Corning, NY, USA) at 10% fetal bovine serum (FBS, Gibco-Life Technologies, Monza, Italy). The solution obtained from the enzymatic digestion was centrifuged at 1200 rpm for 10 min. Then, the pellet obtained was transferred into a T25 culture flask with low-glucose (1 g/L) DMEM supplemented with 10% FBS, 1% antibiotics (100 µg/mL^−1^ streptomycin and 100 IU/mL^−1^ penicillin), and 1% l-glutamine to promote a final spontaneous migration of the cells. The isolated hOBs were cultured at 5% CO_2_ and 37 °C to achieve their confluence to be used between the 3rd and the 5th passage upon the characterization by cytometric analysis.

### Determination of PpIX

To determine the time-dependent intracellular content of PpIX, 6 × 10^3^ cells/well human osteoblasts were plated in 96-well plates and subjected to the ALAD-PDT according to the experimental design. After 0 h, 48 h, and 72 h, cells were treated with a solution of 0.5 M perchloric acid (HClO_4_) in 50% methanol [7] and excited at 405 nm and the PpIX fluorescence was read at 608 nm, by a microplate spectrofluorometer (Synergy H1 Hybrid BioTek Instruments).

### Cell proliferation

The effects of ALAD-PDT on hOB proliferation were assessed by CellTiter96 assay (3-(4,5-dimethylthiazolyl-2)-2,5-diphenyltetrazolium bromide) (MTS, Promega, Madison, WI, USA) at 48 and 72 h. Briefly, 6 × 10^3^ cells/well were seeded in 96-well plates and incubated with ALAD (10%, 50%, 100% v/v) and irradiated by LED according to the experimental design. After 48 and 72 h, cell culture was supplemented with 10 µL of MTS solution and incubated for 2 h. The spectrophotometric absorbance in terms of optical density (OD) was detected at 490 nm by a microplate spectrophotometer (Synergy H1 Hybrid BioTek Instruments). Number of cells was determined in correlation to the OD values. Cell proliferation rate was calculated as a percentage respect to the control.

### ALP activity

The function of osteoblasts after (%)-ALAD-PDT was assessed by determining the alkaline phosphatase (ALP) activity in agreement with the protocol of assay kit (Abcam Inc., Cambridge, UK) on the base of the cleavage of p-nitrophenyl phosphate (pNPP). In brief, 5 × 10^4^ cells/well osteoblasts in 24-well plates were subjected to (%)-ALAD-PDT protocol. After 3 days, the cells were washed three times with PBS and resuspended in assay buffer. The cell suspension was then homogenized through Tissue Rupture device (QIAGEN, Hilden, Germany) and centrifuged at 10,000 g for 15 min. The relative ALP activity of the supernatant was measured using pNPP, as the substrate, for 1 h. After incubation, the reaction was stopped and the product, p-nitrophenol, was quantified as the absorbance value at 405 nm.

### Alizarin Red staining

The effects of ALAD concentrations in combination with LED on the mineralization capability of hOBs were analyzed through Alizarin Red staining (ARS). Untreated and unexposed cells were considered control for comparative evaluation. Cells were seeded at a density of 5 × 10^4^ cells/well onto the surface of the cover slips (Thermo Fisher, Waltham, MA, USA) into the 24-well culture plate. After 14 days of culture, samples were rinsed three times with PBS and fixed with glutaraldehyde solution (2.5%) for 2 h. After fixation, 1 mL of ARS solution (2,003,999, Sigma-Aldrich) was added. After 1 h, the deionized water was used to remove the excess dye, and the presence of mineral nodule stained by red color was observed.

### Quantification of calcium deposition

The quantification of calcium deposition was measured by the addition of the cetylpyridinium chloride (CPC) to the cover slips after the qualitative measurement by ARS, as described in the previous paragraph. A total of 1 mL of 10% CPC solution (Sigma-Aldrich) was added to chelate calcium ions. After 1 h, the absorbance was taken over at 540 nm in a microplate reader (OD540) (Synergy H1 Hybrid BioTek Instruments) and normalized with cell number.

### Statistical analysis

All experiments were performed in biologic triplicates and repeated three times. The data are reported as means ± standard deviation (SD). Statistical analyses were performed using the GraphPad Prism8 (GraphPad Software San Diego California, USA). The ANOVA and post hoc Tukey tests were adopted. A *p* value < 0.05 was considered significant.

## Results

### Levels of PpIX fluorescence

**Fig. 1:**
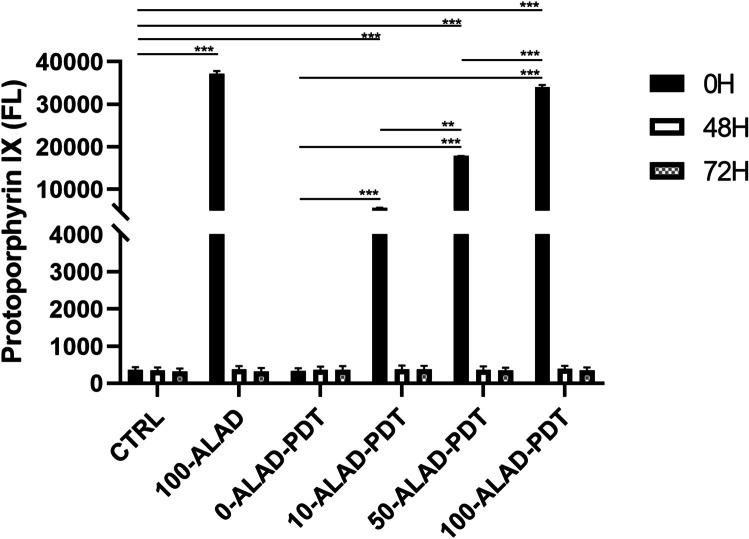
Time-dependent fluorescence of PpIX was measured (*λ*ex = 405 nm; *λ*em = 608 nm) at 0 h, 48 h, and 72 h after ALAD-PDT protocol. Data are presented as mean ± SD of three independent experiments and are expressed in relation of the untreated and unexposed cells (CTRL). (In the graph, the *p* values obtained from the Tukey test are reported, performed after the ANOVA test, **p < 0.001, ***p < 0.0001)

The time-dependent level of PpIX fluorescence was investigated at 0 h, 48 h, and 72 h (Fig. 1). As the experimental design, osteoblasts underwent different concentrations of ALAD gel for 45 min. An increase in the fluorescence of PpIX was observed just immediately after the incubation time of 45 min (0 h) at all concentrations (10, 50, 100% v/v). 100-ALAD determined the highest peak of PpIX fluorescence that slightly dropped down after irradiation. The lower concentrations (10%, 50%) also showed an increment of PpIX fluorescence that was directly proportional to the concentrations, but less than 100%. The fluorometrical measurements at 48 h and 72 h of PpIX resulted to have no differences with respect to the control group. The ANOVA test revealed a *p* < 0.0001.

### ALAD-PDT promoted cell proliferation

**Fig. 2:**
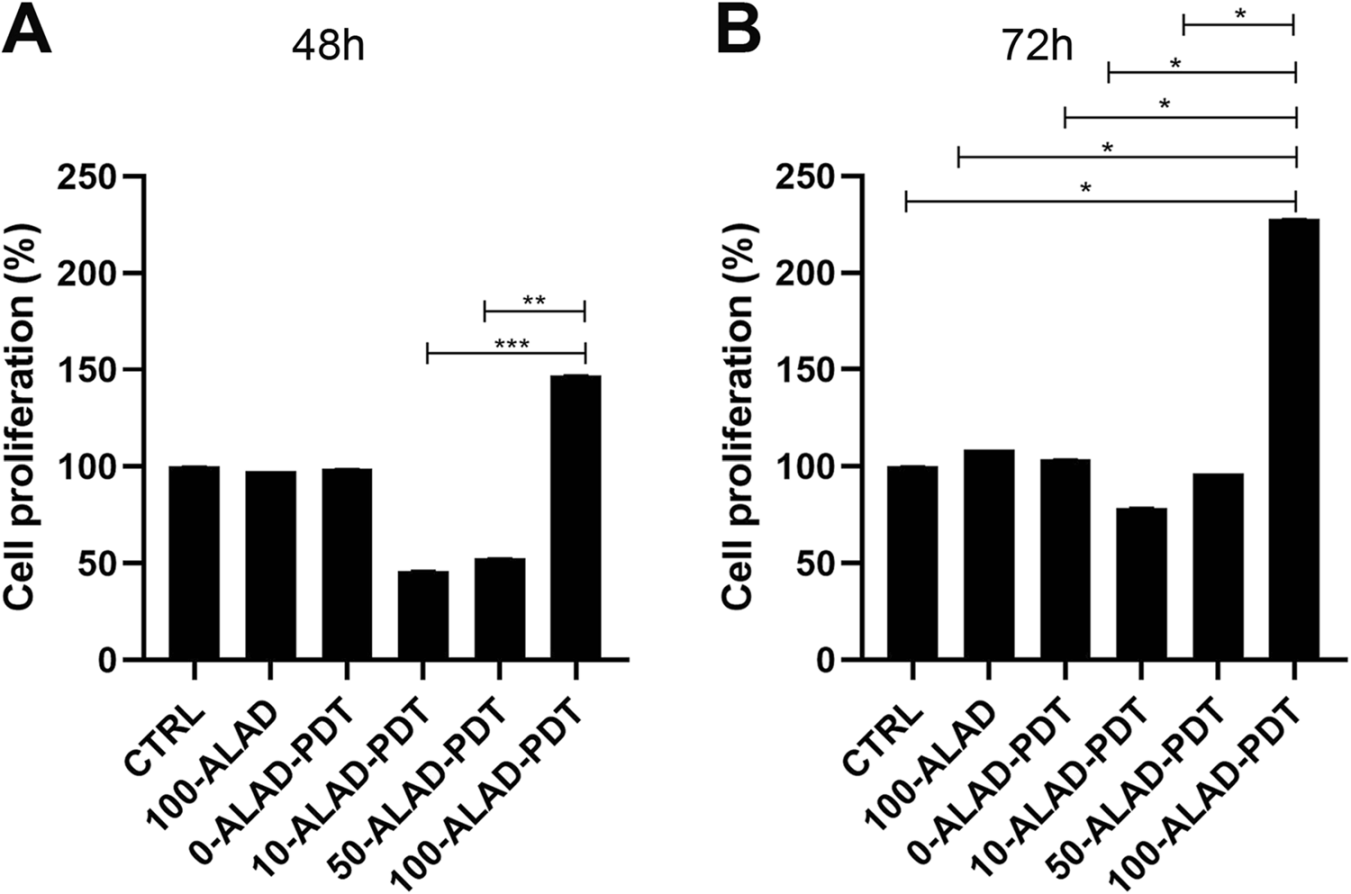
Sensitiveness of osteoblast proliferation rate to different concentrations of ALAD (%) in combination with LED irradiation was determined by the MTS assay at 48 h (**A**) and 72 h (**B**). Data are presented as mean ± SD of three independent experiments in triplicate and are expressed relative to untreated and unexposed cells (CTRL). (In the graphs, the *p* values obtained from the Tukey test are reported, performed after ANOVA test, *p < 0.0461, **p < 0.001, ***p < 0.0001)

The results (Fig. 2) showed a similar trend at both evaluation times 48 h (A) and 72 h (B). Cells subjected to LED exposure alone (0-ALAD-PDT), as well as cells treated with gel without irradiation (100-ALAD), presented the same proliferation rate as control. On the contrary, lower concentrations (10%, 50%) had no appreciable effects on osteoblast proliferation that resulted lower than control. A significant increased in cell proliferation was observed only in osteoblasts subjected to ALAD (100%) and LED. In particular, 100-ALAD-PDT enhanced cell proliferation of + 46.833% ± 0.230 at 48 h and + 127.756% ± 0.154 at 72 h compared to the CTRL. The ANOVA test revealed a *p* = 0.0016 and *p* = 0.0252 at 48 and 72 h, respectively.

### ALAD-PDT stimulated ALP activity

**Fig. 3:**
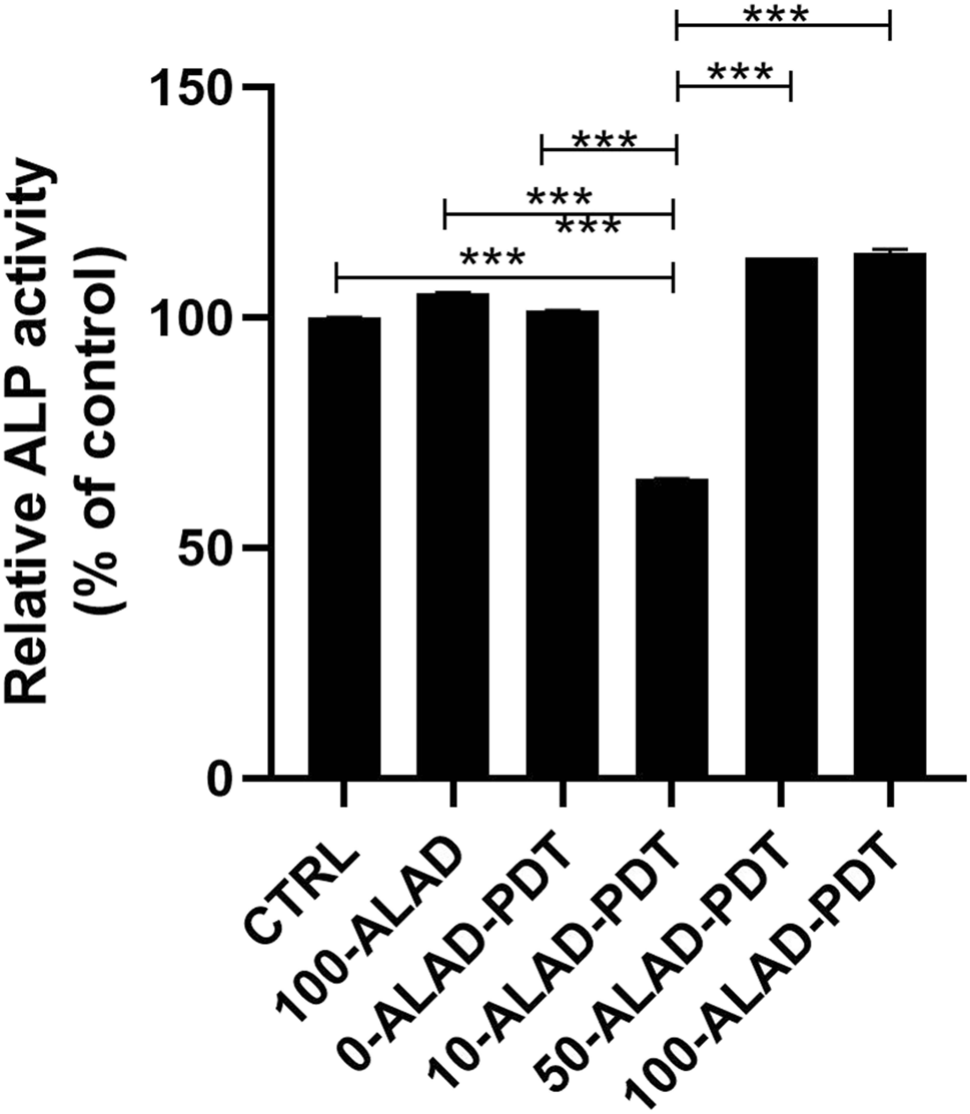
ALP activity of human osteoblasts cultured after 3 days of the ALAD-PDT treatment. ALP activity was increased by ALAD gel used at concentrations of 50% and 100% in combination to the red light, irradiated for 7 min, + 12.910% ± 0.154 and + 14.014% ± 0.146% respectively. Data are expressed with relation to untreated and unexposed cells (CTRL) as mean ± SD. (In the graph, the *p* values obtained from the Tukey test are reported, performed after ANOVA test, ***p < 0.0001)

Results (Fig. 3) showed that the ALP activity, evaluated at 3 days of culture, was slightly increased with respect to control when osteoblasts were exposed to LED alone (0-ALAD-PDT). Similarly, cells treated with ALAD (100%) without irradiation showed the same ALP activity observed in the control, whereas the lowest concentration of ALAD (10%) showed a significant decrease of ALP activity compared to the control. Among the concentrations, both 50- and 100-ALAD in combination of red light irradiation exhibited stimulatory effects on osteoblastic ALP activity. However, this observed increment was not statistically significant with respect to the CTRL.

### ALAD-PDT stimulated mineral deposition

**Fig. 4:**
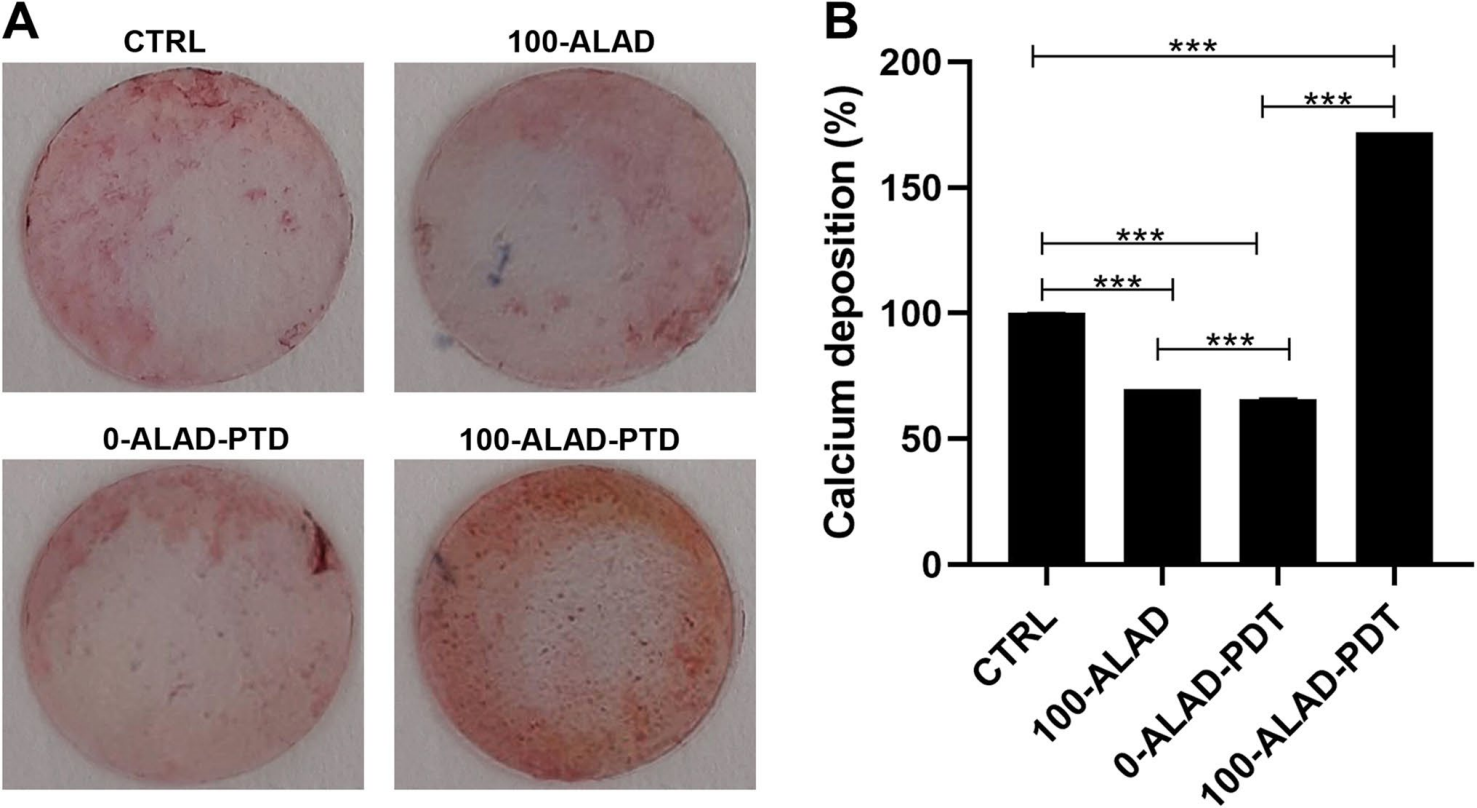
Effects of ALAD-PDT on osteoblast mineralization capacity at day 14. **A** Extracellular matrix calcium deposits were stained with Alizarin Red staining to point out the mineralized nodules. **B** Quantitative measurement was performed with cetylpyridinium chloride. (In the graph, the *p* values obtained from the Tukey test are reported, performed after ANOVA test, ***p < 0.0001)

Beyond the osteoblast proliferation and the ALP activity, the extracellular matrix mineralization is also an important aspect of bone formation. Therefore, osteoblasts were examined at 14 days of culture to assess whether the ALAD-PDT protocol stimulated matrix mineralization, which occurs within the ranging period of 10–15 days and is correlated to an enhanced anabolic activity in bone metabolism. 100-ALAD followed by LED irradiation showed calcified nodules that appeared brighter red after the Alizarin Red staining than osteoblasts only exposed to LED or treated only with 100-ALAD (Fig. 4A). The quantitative results, obtained by CPC experiment, showed that mineralization occurred in the presence of ALAD (100%) and resulted significantly enhanced compared to the control. More definitely, an increase of + 72.033% ± 0.320 in the mineral deposition for 100-ALAD-PDT was measured. In contrast, in the 0-ALAD-PDT, meaning that cells were exposed to LED alone, calcium deposition resulted significantly diminished with respect to the control group (CTRL), indicating that the exposure to LED failed to exert a positive influence on cell mineralization, without ALAD. Similarly, when osteoblasts were treated with 100-ALAD alone, a significant decrease of calcium deposition was observed (Fig. 4B). The ANOVA test revealed a *p* < 0.0001.

## Discussion

The aim of this study was to evaluate the effects on osteoblast response to ALAD-PDT, a protocol that already provides in vitro and in vivo efficacy against pathogens involved in periodontal diseases [4, 16, 22, 23]. Therefore, the PpIX fluorescence, the proliferation, the ALP activity, and bone mineralization capabilities of human oral osteoblasts subjected to ALAD-PDT were examined. In the present study, ALAD alone (100-ALAD) promoted a temporary increase of intracellular PpIX fluorescence just immediately 45 min of gel incubation, returning to the baseline level at 48 h and 72 h. The heme biosynthetic pathway explains this temporarily boosting generation of PpIX [27]. The addition of exogenous aminolevulinic acid can override the negative feedback control normally exerted by a high intracellular concentration of heme on its physiologic biosynthetic pathway, promoting the temporarily rise of the PpIX production. The intervention of the ferrochelatase enzyme in the heme synthesis leads to the observed transient accumulation of PpIX. The light activation of the sample preincubated 45 min with ALAD led to a slight decrease of PpIX fluorescence with respect to the same not-irradiated samples. In particular, the measured fluorescence was proportional to the applied ALAD concentrations (10, 50, 100% v/v). The irradiation caused only a slight drop down of PpIX fluorescence because the immediate effect of light activation leads to the photobleaching of PpIX with the formation of other photoproducts that characterized their self by a similar fluorescence [28, 29]. Rotomskis and co-workers demonstrated that in a PBS solution, hematoporphyrin-like sensitizers are under a dynamic equilibrium between aggregated and monomeric forms. The irradiation of aggregated sensitizers leads to the formation of two distinct photoproducts with a *λ*max at 660 nm [30]. More recently, Huntosova et al. showed that it is not possible to excite specifically PpIX while preventing the excitation of its photoproducts [31]. Results in this study showed that osteoblasts treated with 100-ALAD-PDT presented at 48 h and 72 h a statistically significant increment of the proliferation rate with respect to the control. In contrast, the diluted concentrations of ALAD (10% and 50%) (v/v) did not influenced the osteoblast proliferation. As widely reported in the literature, the other important factor in the PDT therapy is the light at a specific wavelength to activate the photosensitizer molecule. Findings from this present study showed that cells exposed to LED alone without the addition of any concentrations of ALAD had no effects on cell proliferation compared to unexposed osteoblasts. Thus, the involvement of both light source and photosensitizer molecules is required to obtain an efficient PDT performance. The enhanced osteoblast proliferation by 100-ALAD-PDT indicating a promotion of cell growth rate may suggest a stimulating effect on the bone matrix formation. Therefore, the alkaline phosphatase (ALP) activity that represents the most widely studied biochemical marker for osteoblastic activity was also assessed. 100-ALAD-PDT treatment also promoted cellular ALP activity, which has been measured at day 3. Although the increased ALP activity in cells was observed, it was not statistically significant. It could be speculated that ALP activity increases in relation to the temporal growth and differentiation of osteoblasts, ranging from 5 to 15 days immediately after the cell proliferation period which occurs from 2 to 5 days [32]. This would imply that at the time of our analysis, which is the third day, the ALP activity has just started. On the other hand, matrix mineralization is preceded by the osteoblast differentiation phase and occurs within the ranging period of 10–15 days. In the present study, we expected to note calcium deposits around day 14, and at that time, the measurement of extracellular matrix calcium deposits indicated a significant positive effect of 100-ALAD in combination with LED, on calcification. Increased calcium deposition in extracellular matrix, when cells were treated with ALAD following red light exposure (100-ALAD-PDT), was then confirmed by Alizarin Red staining, which indicated stimulation of mineralization. This could be explained by the ability of 5-delta aminolevulinic acid to upregulate heme oxygenase 1 (HO-1) as shown in recent studies [33, 34]. HO-1 is involved in the normal physiological bone homeostasis, by promoting bone mesenchymal stem cell differentiation, by enhancing osteoblasts functions, and by inhibiting apoptosis and senescence of osteoblasts [35]. In this study, LED irradiation alone did not favor the mineralized nodule formation as well as the 100-ALAD treatment. Although studies reported the topical use of 5-delta aminolevulinic acid as a pro-drug, to cure many diseases, like pre-cancer conditions, to date few investigations have been performed to explore a potential regenerative effect of ALAD-PDT. Egli RJ et al. in 2007 observed a reduction of viable dendritic, fibroblast, and osteoblast cells, when incubated with 5-delta aminolevulinic acid for 4 h and subjected to increasing light doses [25]. Bastian JD et al. in 2009 showed a decrease of viability of porcine chondrocytes and osteoblasts after the incubation with 5-ala for 4 h and exposed to light doses up to 20 J/cm^2^ [24]. Our findings might suggest that a different approach in terms of gel incubation and light exposing times can exert very encouraging results. Despite available evidence on the use of ALAD gel in treating chronic inflammatory skin diseases are scanty, a recent study showed that concentration of aminolevulinic acid (5%) present into gel is better tolerated by human cells than higher concentrations (i.e., 20%) [21]. A strength of this study is the use of primary human cells which represent a good system to study biochemical signaling in vivo closer to human state than mouse and rat cells used in the previous literature [26]. The osteoblasts used in this study have been extracted from oral biopsies because the tested protocol ALAD-PDT is manufactured for treating periodontitis and peri-implantitis that can lead to a progressive bone loss. Therefore, more data need to be carried out to show the extent of the utility of ALAD-PDT, beyond the antimicrobial strategy. Further studies will be necessary to clarify the mechanism that leads the 5-delta aminolevulinic acid in combination with LED to influence osteogenic functions, and to investigate for expanding our knowledge about the stimulatory role of ALAD on bone cells.

## Conclusions

These findings suggest that osteoblastic cells incubated for 45 min with ALAD gel as commercialized and exposed for 7 min to the red light of 630 nm LED showed a statistical increase of cell growth, and a stimulatory effect on ALP activity and mineralization.

## Declarations

This study has been approved by the Ethics Committee for Biomedical Research of Chieti and Pescara District and University Gabriele d’Annunzio Chieti-Pescara (protocol code BONEISTO N. 22 10 July 2021). The procedures used in this study adhere to the tenets of the Declaration of Helsinki.

## References

[1] D’Ercole S, Spoto G, Trentini P (2016). In vitro inactivation of Enterococcus faecalis with a led device. J Photochem Photobiol B.

[2] Petrini M, Trentini P, Tripodi D (2017). In vitro antimicrobial activity of LED irradiation on Pseudomonas aeruginosa. J Photochem Photobiol B.

[3] Petrini M, Spoto G, Scarano A (2019). Near-infrared LEDS provide persistent and increasing protection against E. faecalis. J Photochem Photobiol B.

[4] Lauritano D, Moreo G, Palmieri A (2022). Photodynamic therapy using 5-aminolevulinic acid (Ala) for the treatment of chronic periodontitis: a prospective case series. Appl Sci.

[5] Kwiatkowski S, Knap B, Przystupski D (2018). Photodynamic therapy - mechanisms, photosensitizers and combinations. Biomed Pharmacother = Biomed Pharmacother.

[6] Collaud S, Peng Q, Gurny R, Lange N (2008). Thermosetting gel for the delivery of 5-aminolevulinic acid esters to the cervix. J Pharm Sci.

[7] Moan J, Bech Ø, Gaullier JM, et al. (1998) Protoporphyrin IX accumulation in cells treated with 5-aminolevulinic acid: dependence on cell density, cell size and cell cycle. Int J Cancer 75. 10.1002/(SICI)1097-0215(19980105)75:1<134::AID-IJC20>3.0.CO;2-F 10.1002/(sici)1097-0215(19980105)75:1<134::aid-ijc20>3.0.co;2-f 9426701

[8] Egli RJ, di Criscio A, Hempfing A (2008). In vitro resistance of articular chondrocytes to 5-aminolevulinic acid based photodynamic therapy. Lasers Surg Med.

[9] Sculean A, Deppe H, Miron R (2021). Effectiveness of photodynamic therapy in the treatment of periodontal and peri-implant diseases. Monogr Oral Sci.

[10] Chambrone L, Wang HL, Romanos GE (2018). Antimicrobial photodynamic therapy for the treatment of periodontitis and peri-implantitis: an American Academy of Periodontology best evidence review. J Periodontol.

[11] Alwaeli HA, Al-Khateeb SN, Al-Sadi A (2015). Long-term clinical effect of adjunctive antimicrobial photodynamic therapy in periodontal treatment: a randomized clinical trial. Lasers Med Sci.

[12] Elsadek MF, Farahat MF (2022) Impact of photodynamic therapy as an adjunct to non-surgical periodontal treatment on clinical and biochemical parameters among patients having mild rheumatoid arthritis with periodontitis. Photodiagnosis Photodyn Ther 37. 10.1016/J.PDPDT.2021.102698 10.1016/j.pdpdt.2021.102698 34921986

[13] Li Q, Jiao B, Zhou F (2014). Comparative study of photodynamic therapy with 5%, 10% and 20% aminolevulinic acid in the treatment of generalized recalcitrant facial verruca plana: a randomized clinical trial. J Eur Acad Dermatol Venereol.

[14] Gallagher-Colombo SM, Quon H, Malloy KM (2015). Measuring the physiologic properties of oral lesions receiving fractionated photodynamic therapy. Photochem Photobiol.

[15] Lei X, Liu B, Huang Z, Wu J (2015). A clinical study of photodynamic therapy for chronic skin ulcers in lower limbs infected with Pseudomonas aeruginosa. Arch Dermatol Res.

[16] Radunović M, Petrini M, Vlajic T, et al. (2020) Effects of a novel gel containing 5-aminolevulinic acid and red LED against bacteria involved in peri-implantitis and other oral infections. J Photochem Photobiol B 205. 10.1016/J.JPHOTOBIOL.2020.111826 10.1016/j.jphotobiol.2020.111826 32146270

[17] Greco G, di Piazza S, Chan J, et al. (2020) Newly formulated 5% 5-aminolevulinic acid photodynamic therapy on Candida albicans. Photodiagnosis Photodyn Ther 29. 10.1016/J.PDPDT.2019.10.010 10.1016/j.pdpdt.2019.10.010 31614222

[18] Lotufo MA, Tempestini Horliana ACR, Santana T, et al. (2020) Efficacy of photodynamic therapy on the treatment of herpes labialis: a systematic review. Photodiagnosis Photodyn Ther 29. 10.1016/J.PDPDT.2019.08.018 10.1016/j.pdpdt.2019.08.018 31648056

[19] Jeong B, Kim SW, Bae YH (2002). Thermosensitive sol-gel reversible hydrogels. Adv Drug Deliv Rev.

[20] Dumortier G, Grossiord JL, Agnely F, Chaumeil JC (2006). A review of poloxamer 407 pharmaceutical and pharmacological characteristics. Pharm Res.

[21] Serini SM, Cannizzaro MV, Dattola A (2019). The efficacy and tolerability of 5-aminolevulinic acid 5% thermosetting gel photodynamic therapy (PDT) in the treatment of mild-to-moderate acne vulgaris. A two-center, prospective assessor-blinded, proof-of-concept study. J Cosmet Dermatol.

[22] Petrini M, di Lodovico S, Iezzi G, et al. (2022) Photodynamic antibiofilm and antibacterial activity of a new gel with 5-aminolevulinic acid on infected titanium surfaces. Biomedicines 10. 10.3390/BIOMEDICINES10030572 10.3390/biomedicines10030572 PMC8945072 35327374

[23] Petrini M, Pierfelice TV, D’amico E, et al. (2022) Comparison between single and multi-LED emitters for photodynamic therapy: an in vitro study on Enterococcus faecalis and human gingival fibroblasts. Int J Environ Res Public Health 19. 10.3390/IJERPH19053048 10.3390/ijerph19053048 PMC8910628 35270740

[24] Bastian JD, Egli RJ, Ganz R (2009). Differential response of porcine osteoblasts and chondrocytes in cell or tissue culture after 5-aminolevulinic acid-based photodynamic therapy. Osteoarthritis Cartilage.

[25] Egli RJ, Schober M, Hempfing A (2007). Sensitivity of osteoblasts, fibroblasts, bone marrow cells, and dendritic cells to 5-aminolevulinic acid based photodynamic therapy. J Photochem Photobiol B.

[26] Kushibiki T, Tu Y, Abu-Yousif AO, Hasan T (2015) Photodynamic activation as a molecular switch to promote osteoblast cell differentiation via AP-1 activation. Sci Rep 5. 10.1038/SREP13114 10.1038/srep13114 PMC4538568 26279470

[27] Obi CD, Bhuiyan T, Dailey HA, Medlock AE (2022) Ferrochelatase: mapping the intersection of iron and porphyrin metabolism in the mitochondria. Front Cell Dev Biol 10. 10.3389/FCELL.2022.894591 10.3389/fcell.2022.894591 PMC9133952 35646904

[28] Bonnett R, Martínez G (2001). Photobleaching of sensitisers used in photodynamic therapy. Tetrahedron.

[29] Finlay JC, Conover DL, Hull EL, Foster TH (2001). Porphyrin bleaching and PDT-induced spectral changes are irradiance dependent in ALA-sensitized normal rat skin in vivo¶. Photochem Photobiol.

[30] Rotomskis R, Bagdonas S, Streckyte G (1996). Spectroscopic studies of photobleaching and photoproduct formation of porphyrins used in tumour therapy. J Photochem Photobiol B.

[31] Huntosova V, Gerelli E, Zellweger M, Wagnières G (2016). Effect of PpIX photoproducts formation on pO 2 measurement by time-resolved delayed fluorescence spectroscopy of PpIX in solution and in vivo. J Photochem Photobiol B.

[32] Lian JB, Stein GS (1992). Concepts of osteoblast growth and differentiation: basis for modulation of bone cell development and tissue formation. Crit Rev Oral Biol Med.

[33] Liu C, Zhu P, Fujino M (2019). 5-aminolaevulinic acid (ALA), enhances heme oxygenase (HO)-1 expression and attenuates tubulointerstitial fibrosis and renal apoptosis in chronic cyclosporine nephropathy. Biochem Biophys Res Commun.

[34] Ito H, Nishio Y, Hara T (2018). Oral administration of 5-aminolevulinic acid induces heme oxygenase-1 expression in peripheral blood mononuclear cells of healthy human subjects in combination with ferrous iron. Eur J Pharmacol.

[35] Zhou X, Yuan W, Xiong X, et al. (2021) HO-1 in bone biology: potential therapeutic strategies for osteoporosis. Front Cell Dev Biol 9. 10.3389/FCELL.2021.791585 10.3389/fcell.2021.791585 PMC8669958 34917622

